# Perioperative levetiracetam for seizure prophylaxis in seizure-naive brain tumor patients with focus on neurocognitive functioning

**DOI:** 10.1186/s12883-022-02762-7

**Published:** 2022-07-08

**Authors:** Elias Konrath, Franz Marhold, Wolfgang Kindler, Florian Scheichel, Branko Popadic, Katrin Blauensteiner, Bernadette Calabek, Elisabeth Freydl, Michael Weber, Robin Ristl, Katharina Hainz, Camillo Sherif, Stefan Oberndorfer

**Affiliations:** 1 Karl Landsteiner University of Health Sciences, Dr. Karl-Dorrek-Straße 30, 3500 Krems, Austria; 2 Department of Neurology, University Hospital St. Pölten, Dunant-Platz 1, 3100 St. Pölten, Austria; 3 Department of Neurosurgery, University Hospital St. Pölten, Dunant-Platz 1, 3100 St. Pölten, Austria; 4 Department of General Health Studies, Division Biostatistics and Data Science, Karl Landsteiner University of Health Sciences, Dr. Karl-Dorrek-Straße 30, 3500 Krems, Austria; 5 Section for Medical Statistics, Center for Medical Statistics, Informatics and Intelligent Systems, Medical University of Vienna, Spitalgasse 23, 1090 Vienna, Austria; 6 Karl Landsteiner Institute for Clinical Neurology and Neuropsychology, c/o Department Neurology, 3100 St. Pölten, Austria

**Keywords:** Cognitive functioning, Cognition, Brain tumor, Perioperative seizure prophylaxis, Surgery

## Abstract

**Introduction:**

In seizure-naive brain tumor patients, the efficacy of perioperative prophylactic antiepileptic drug treatment remains controversial. In case of administration, the common preferred drug is levetiracetam (LEV) because of its favorable pharmacological profile. Research to date has not sufficiently determined how LEV affects cognition in the short term, as is the case in the perioperative period. The objective of this prospective study was to examine the neurocognitive functioning of seizure-naive brain tumor patients after receiving LEV perioperatively.

**Methods:**

Fortythree patients with supratentorial brain tumor scheduled for surgery received LEV three days before until six days after surgery as seizure prophylaxis. Cognitive functioning (NeuroCogFX), LEV plasma-levels, hematotoxicity, side-effects, as well as health-related quality of life (HRQoL, Qolie31), were recorded preoperatively before (Baseline) and after onset of LEV (Pre-Op), 4–6 days postoperatively (Post-Op) and 21 days postoperatively (Follow-Up).

**Results:**

No significant changes in cognitive functioning and HRQoL were seen after onset of preoperative LEV. There was a significant improvement of NeuroCogFX total-score at Follow-Up (*p* = 0.004) compared to Baseline. The overall-score Qolie31 showed simultaneous improvement patterns as cognitive functioning (*p* < 0.001). The most frequent side effect related to study drug was somnolence (in 28.6% of patients).

**Conclusions:**

A significant improvement of cognitive functioning, as well as an improvement in HRQoL, were detected postoperatively. This is presumably due to the debulking effect of the surgery. Nevertheless, LEV has no detrimental effect on cognitive functioning in the perioperative phase in seizure-naive brain tumor patients.

**Trial registration:**

This study was registered prospectively (Date: 25/11/2015; EudraCT: 2015–003,916-19).

**Supplementary Information:**

The online version contains supplementary material available at 10.1186/s12883-022-02762-7.

## Introduction

The efficacy of prophylactic antiepileptic drug (AED) administration in seizure-naive brain tumor patients remains controversial [1, 2]. Although practice guidelines released by the American Academy of Neurology in 2000 discouraged its use [3], the administration of AEDs for preventing perioperative seizures has been reported to be a common practice by most surveyed neurosurgeons [4, 5]. Nowadays, attempts are made to identify patients with an increased risk profile, considering factors such as tumor location, tumor grade, molecular pathology, and histology. [1, 6–10] 

The incidence of perioperative seizures in seizure-naive brain tumor patients was typically reported as 5 to 10% in most studies [11–16]. Perioperative seizures are associated with longer hospitalization, reduced quality of life, decreased overall survival, increased morbidity, and enhanced risk for development of epilepsy [14, 15]. When perioperative prophylactic AEDs are administered, the current preferred drug is levetiracetam (LEV), which is superior to older AEDs in terms of pharmacokinetics-, tolerability-, safety- and interaction profile, as well as considering the potential synergistic effect on oncologic treatment [1, 8, 9, 17–19]. There is sufficient evidence that the usage of older AEDs—like phenytoin, carbamazepine or valproate—can result in serious adverse effects and interfere with the metabolism of oncologic treatments and anesthetics [1, 3, 7, 12, 20]. Side effects of LEV are generally infrequent, mild, and predominantly of psychiatric nature, with somnolence, asthenia, mood, and behavior problems being the most common in brain tumor patients [1, 19, 21–24].

Discontinuations due to LEV-related adverse effects are uncommon, especially when compared to the discontinuation’s rate in studies performed with older AEDs [12, 13, 18, 24]. Clinical trials in healthy patient populations treated with first-generation AEDs have shown that major adverse effects and reasons for discontinuation were alterations in cognitive functioning [25, 26], the most commonly affected cognitive domains being attention, psychomotor speed and memory [26]. Since cognitive impairment has a great impact on HRQoL, it is important to preserve and restore cognitive functioning [27]. As brain tumor patients often already experience impaired cognitive functioning and restricted HRQoL associated with the tumor, the treatment, and patient-related factors, it is crucial to avoid an additional burden of cognitive side effects related to AED use [28, 29]. In contrast, LEV has been reported not to promote detrimental effects on cognition in epilepsy patients [8, 30, 31]. Moreover, some studies suggest that LEV shows an improvement in a range of cognitive abilities, as well as a potential neuroprotective effect [23, 26, 32–34]. This was described not only in patients with general epilepsy, but also in patients with brain tumor-related epilepsy or even in healthy subjects[35].

In perioperative seizure prophylaxis, it is common for a large proportion of patients to be administered LEV one week postoperatively [3, 5, 36]. However, research to date has not sufficiently determined how LEV affects short-term cognition.

This prospective study was aimed to investigate the short-term effects of perioperatively administered LEV not only on HRQoL, side effects, hematotoxicity, and seizure frequency, but specifically on cognitive functioning in seizure-free brain tumor patients undergoing surgery.

## Methods

### Study population

This study was performed at the Department of Neurology and the Department of Neurosurgery at the University Hospital Sankt Pölten, Austria. This trial included seizure-naive, adult patients (> 18 years) presenting with a radiological suspected primary supratentorial brain tumor and planned surgery. Exclusion criteria comprised a contraindication against LEV and a pre-existing anticonvulsive medication.

### Study design

**Fig. 1:**
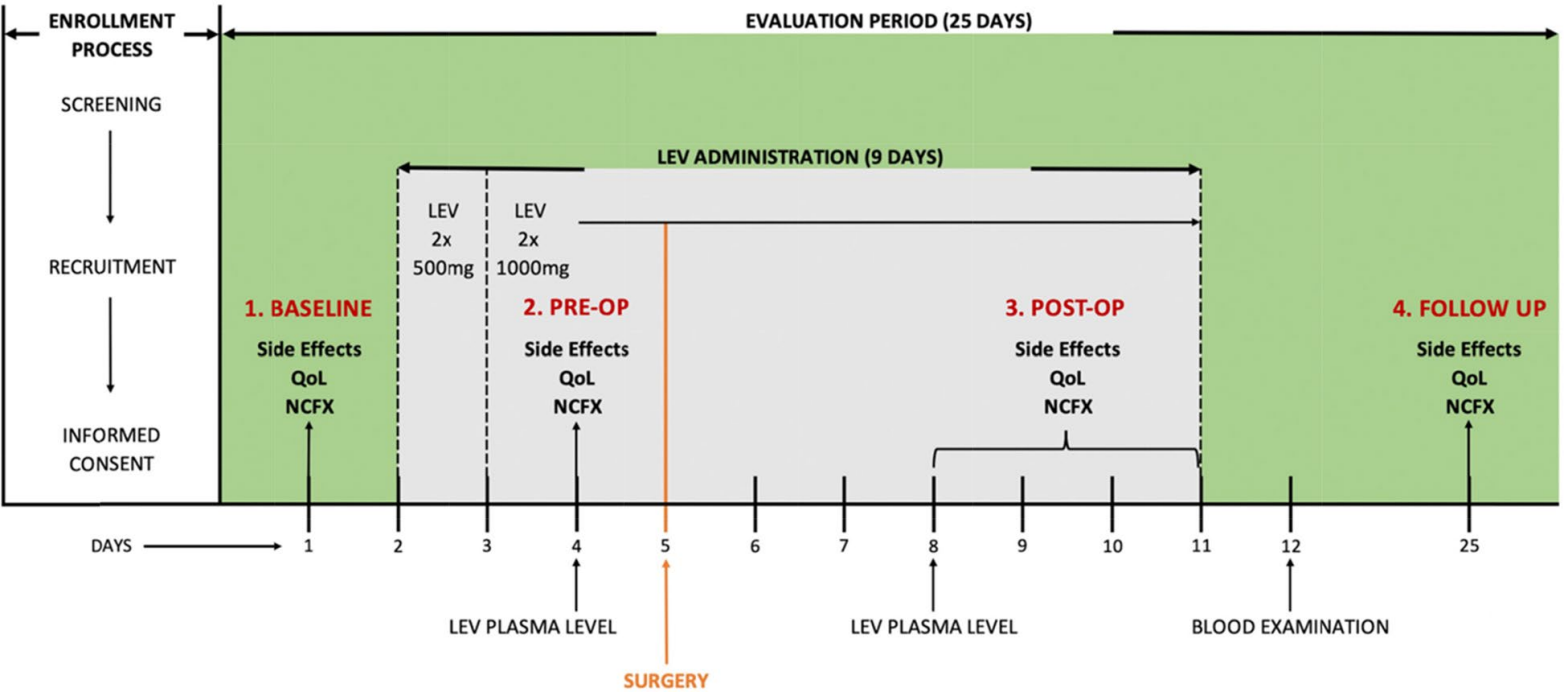
Study Design and Procedures. Patients received LEV during a perioperative period of nine days. Levetiracetam plasma levels were measured two days after onset of LEV administration and three days postoperative. Hematotoxicity was measured through blood samples one week after surgery. Neuropsychological assessment (NeuroCogFX), including HRQoL questionnaire (QOLIE-31) and self-reported side effects, was conducted at four timepoints: one day before administration of levetiracetam (Baseline/no LEV), on the second day after onset of levetiracetam administration (Pre-Op/with LEV), four to six days after surgery (Post-Op/with LEV) and three weeks after surgery (Follow-Up/no LEV). The total study duration was 25 days

Study design is shown in Fig. 1. Patients with a supratentorial brain tumor were administered oral LEV during the perioperative period, ranging from three days before until six days after surgery. The starting dose of LEV (2 × 500 mg on the first day) was escalated to 2 × 1000 mg on the second day and was maintained at this dose for overall nine days. LEV plasma levels were measured two days after first LEV administration and three days after surgery. Hematological markers were measured one week after surgery. Neuropsychological assessments (NeuroCogFX), including HRQoL questionnaire (QOLIE-31) and self-reported side effects, were conducted at four timepoints: one day before administration of levetiracetam (Baseline/no LEV), on the second day after onset of levetiracetam (Pre-Op/with LEV), four to six days after surgery (Post-Op/with LEV) and three weeks after surgery (Follow-Up/no LEV). Every patient received magnetic resonance imaging two days after surgery in a routine matter to exclude postoperative complications such as post-surgical hemorrhage or ischemia and to depict the amount of resection. The total study duration for each patient was 25 days.

### Statistical analysis

﻿Statistical analyses were performed using SPSS-26 software. The significance level was set at alpha = 0.5.

### Cognitive functioning/quality of life over time

A longitudinal Linear Mixed Model (LMM) analysis was conducted to investigate the course of cognitive functioning and HRQoL over time. The categorical factor variable “Time” was specified as repeated. We specified an unstructured correlational structure for the G-matrix. We used restricted maximum likelihood algorithm to estimate model parameters. In the primary analysis, we created models with “Time” as categorical predictor. We performed a separate model for each cognitive score/HRQoL subscale. Due to small sample size, we have not applied correction for multiple testing regarding the multiple models we have performed to investigate all cognitive tests and HRQoL subscales. We performed a Bonferroni adjusted post-hoc analysis to explore the differences between separate timepoints. To examine the connection between cognitive functioning and HRQoL, we performed Pearson correlations for the NeuroCogFX total score and the QOLIE31 overall score for each timepoint.

### Timepoint interval analysis cognitive functioning

To determine the change over the entire evaluation period, we compared cognitive Baseline- and Follow-up performance, both without LEV. To assess potential detrimental effects of LEV on cognitive functioning, we examined changes between Baseline performances without LEV and Pre-Op timepoint with LEV. As the reported critical differences of the cognitive subtests for individual subjects are throughout larger than one standard deviation and considering the recommendations of the authors of NeuroCogFX, we considered a clinically meaningful deterioration of minus ten standard value points as clinically relevant [37]. To examine a potential negative impact, we looked at the uncorrected statistical p-values and compared the lower bound of the corresponding 95% Bonferroni adjusted confidence interval with the meaningful deterioration margin.

### Subgroup analysis neurosurgical procedere

As we had different extents of resection in our sample, we looked if the results from the primary analysis of cognitive data changed once we controlled for the influence of the categorical factor variables “neurosurgical procedere” (biopsy; partial/total resection). We have also included interaction terms with time, to account for possible differences in performance over time.

### Measures

﻿Clinically evident seizure frequency and classification were recorded throughout the whole evaluation period. Self-reported side effects of AEDs were assessed via a questionnaire listing the most frequent side effects, with the option for reporting additional ones [38]. ﻿Patients were asked to rate the presence and strength of impairments on a five-point scale. A senior neurologist evaluated the potential relationship of emerging side effects to the study drug. To measure hematotoxicity, we analyzed the blood concentration of hemoglobin, thrombocytes, leukocytes, and lymphocytes. Hematological toxicity and side effect severity were graded according to the Common Terminology Criteria for Adverse Events (CTCAE), v5.0 [39]. In addition to side effects known from the literature, we have also documented adverse events (AEs). To ensure that LEV was exerting its effect, we collected plasma levels at two timepoints. A possible difference in pre- and postoperative LEV plasma levels was tested for statistical significance using a paired t-test.

### Cognitive functioning (NeuroCogFX)

NeuroCogFX is a computerized neuropsychological screening instrument for serial examinations of patients with epilepsy and brain tumors [37, 40]. Systematic assessment of cognitive function is often restricted to insensitive measures such as the Mini Mental State Examination, whereas conventional neuropsychological testing is time consuming and requires patients willing and able to undergo one to two hours of neuropsychological testing. NeuroCog FX is a compromise between length of the procedure yet comprehensiveness.

Eight subtests address four cognitive domains: attention (Simple Reaction, Go/No-Go, Invers Go/No-Go), working memory (digit span, two back), memory (verbal memory, figural memory) and language (phonematic fluency). Raw scores are converted in age-adjusted standard values (*M* = 100, *SD* = 10, age groups: 16–29, 30–44, 45–59, 60–75 years). Parallel forms were used throughout the study for those available subtests (Phonematic Fluency, Verbal Memory, Figural Memory). Three measures of overall performance are defined: performance scores “Speed” and “Quality” and total score “Total”. An overview of the composition of subtest-, domain-, performance- and Total score can be seen in Supplementary Fig. 1.

### Health-related quality of life (QOLIE31)

HRQoL was assessed with the “Quality of Life in Epilepsy” questionnaire (QOLIE31), which consists of seven subscales [41]. The raw scores are rescaled from zero to 100, with higher values reflecting better HRQoL. An overall score is obtained by summing the subscale scores after weighting using coefficients [41].

## Results

### Study population

**Fig. 2:**
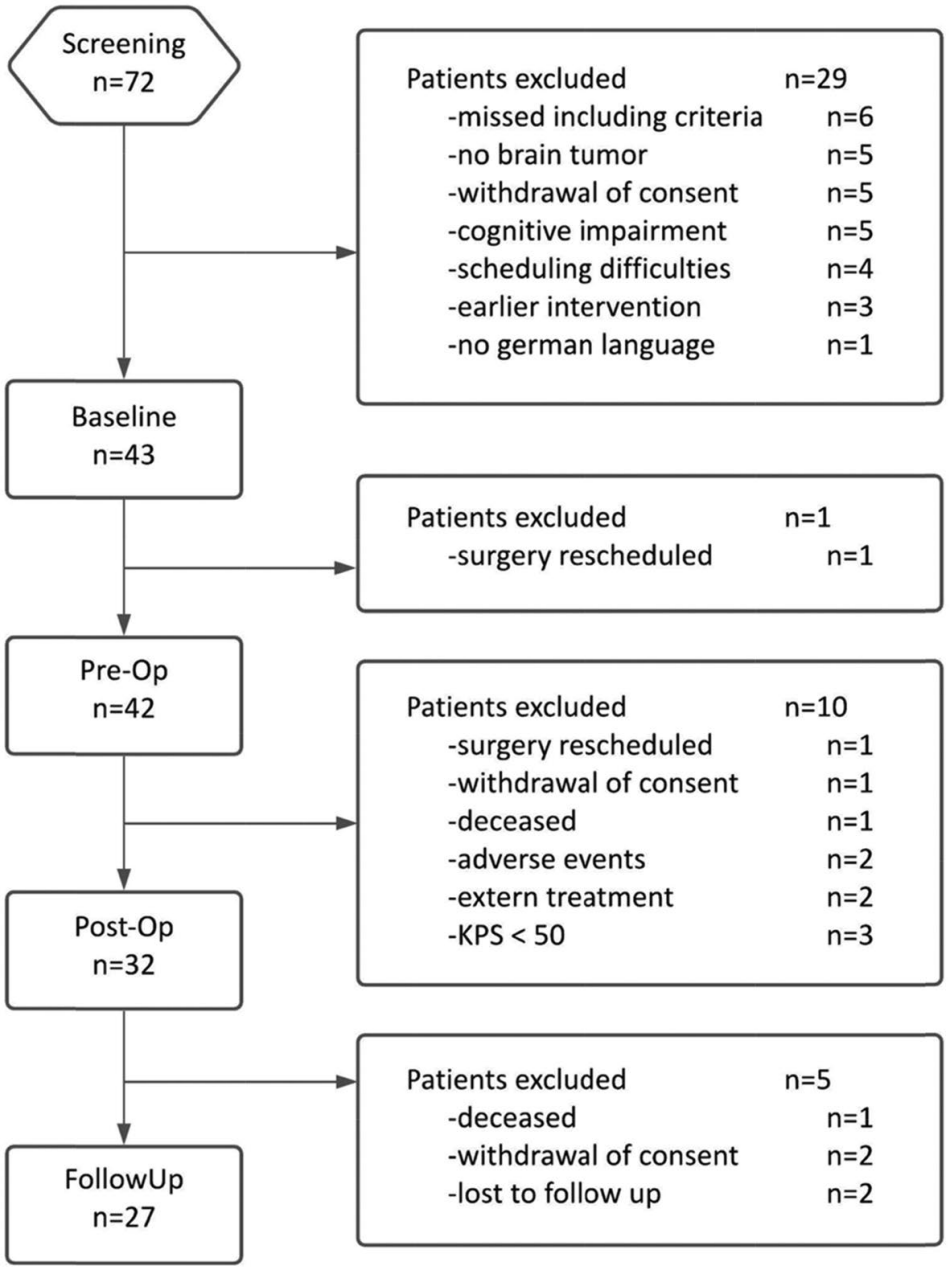
Flow chart of patient inclusion and dropouts. Inclusion criteria comprised seizure-naive, adult patients (> 18 years) with a suspected primary supratentorial brain tumor and a planned surgery. Exclusion criteria included contraindication against LEV and pre-existing anticonvulsive medication

As shown in Fig. 2, 72 patients were screened between February 2016 and May 2020. A total of 141 neuropsychological tests were performed by the 43 eligible subjects. Twenty-seven patients completed all four timepoints as defined per protocol. Demographics and clinical characteristics are summarized in Table 1.

**Table 1:** Frequencies of patient-, tumor- and intervention variables

	***M***	***Range***
**Age (years)**	**60.6**	**33.5 – 82.8**
**Patients**	***n***	***%***
Number of included patients	43	100
Gender		
Male	18	41.9
Female	25	58.1
Tumor grade ^a^		
Low Grade (WHO I&II)	11	25.6
High Grade (WHO III&IV)	27	62.8
No WHO classification	5	11.6
Histology ^a^		
Malignant Glioma	24	55.8
Meningioma	12	27.9
Brain Metastases	4	9.3
Others	3	7.0
Affected hemisphere		
Left	19	44.2
Right	24	55.8
Tumor location		
Frontal	23	53.5
Temporal	9	20.9
Parietal	6	14
Occipital	4	9.3
Trigonal	1	2.3
Neurosurgical procedere		
Gross total resection	16	37.2
Partial resection	19	44.2
Stereotacted, neuronavigated biopsy	7	16.3
No surgery	1	2.3

Abbreviations: *n* frequency, *%* frequency in percentage, *M* mean

^a^Tumor grading according to World Health Organization (WHO) 2016 classification

### Adverse events

Adverse events occurred in two patients (4.65%), one of them showing postoperative psychotic symptoms and the other one a subarachnoid hemorrhage. One patient was not treated surgically due to the development of sigmoid diverticulitis with perforation.

### Seizure frequency and LEV plasma level

Before surgery, LEV plasma level ranged from 4.80 to 50.51 μg/ml (mean, 18.16 μg/ml) and after surgery from 5.64 to 59.65 μg/ml (mean, 18.15 μg/ml). The laboratory reference value range was 12 to 46 μg/ml. Two implausible values (0 and 0.56 μg/ml) were excluded from the analysis. Paired t-test was not significant (t(29) = 0.541; p = 0.592). Two patients (4.7%) had a seizure three days postoperatively (Day 6 after onset of Lev), one being classified as self-limited focal and the other as suspected complex focal; LEV plasma levels at seizure occurrence were 9.75 and 18.85 μg/ml. Scatterplots of LEV plasma levels for both measurement points are given in Supplementary Fig. 2, while descriptive data are shown in Supplementary Table 1.

### Hematotoxicity

CTCAE-classification of hematotoxicity AEs are given in Supplementary Fig. 3. According to this classification, no hematological AEs were categorized as Grade 4 or reported as treatment-related AEs in any of the patients. Grade 3 AE occurred in lymphocytes only (5.1%). All other AEs were classified as follow: moderate in lymphocytes (10.3%), in hemoglobin (7%) and in thrombocytes (2.3%); mild in thrombocytes (25.6%), in hemoglobin (20.9%) and in lymphocytes (18%); or within the normal range.

### Side effects

A precise breakdown of the frequencies of side effects and severities over the entire study period and for the individual points in time can be found in Supplementary Table 2. Frequency of patients reporting side effects related to study drug levetiracetam in absolute percentage across four timepoints can be found in Supplementary Fig. 4. Across the three timepoints after Baseline, 21 patients (48.8%) reported in total 42 side effects. Distribution of severity was 19 mild, (45.2%); 16 moderate, (38.1%); 6 severe, (14.3%); 1 serious, (2.4%). Subdivided by timepoint, the number of patient-reported side effects was 16 in Pre-Op (37.2%), twelve in Post-Op (27.9%) and seven in Follow-Up (16.7%). Somnolence was the most frequent, as reported by twelve patients (28.6%). Subjective memory impairment and vertigo were reported by three patients (7.1% each), cephalea, depression and exanthem by two patients (4.8% each) and nasopharyngitis, nausea, diarrhea, and ophthalmalgia by one patient (2.4%). None of the patients reported abdominal pain, concentration impairment, amnestic aphasia, aggression, anxiety, nightmare and tinnitus. There were no serious adverse effects related to LEV and no suspected adverse drug reactions.

### Cognitive performance over time

**Fig. 3:**
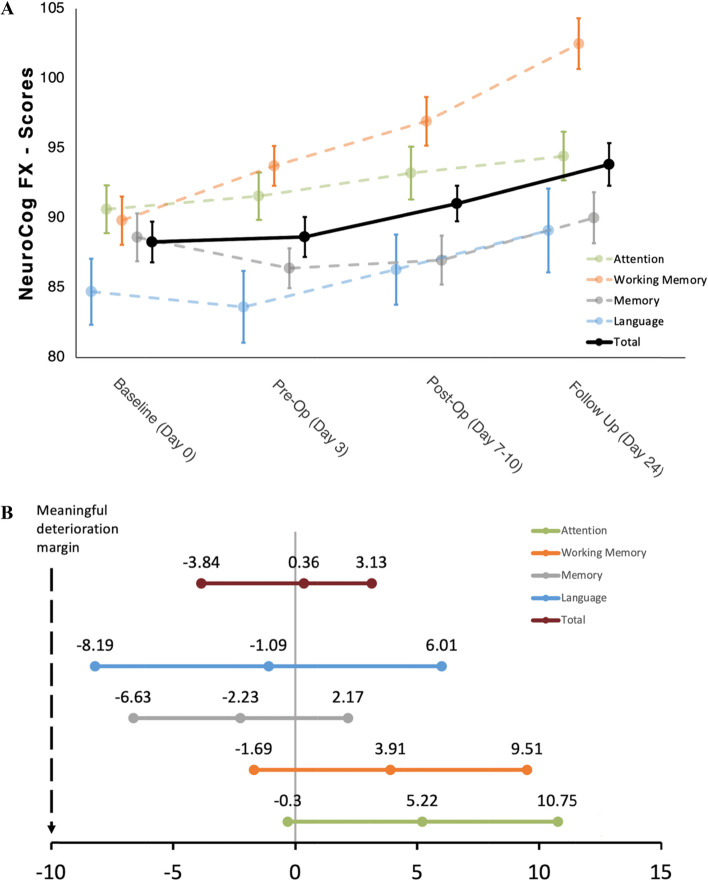
**(A)** Estimated marginal means and corresponding standard error bars in standard value points across the four timepoints for the cognitive domain scores and Total score. Neuropsychological assessment of cognitive functioning was measured at four timepoints (Fig. 1). Total Score consists of the following domains: attention, working memory, memory and language. Which subtests constitute domains, see methods section. **(B)** Estimated marginal mean differences and corresponding 95%, Bonferroni adjusted confidence intervals of standard value points for cognitive domain scores and Total score between Pre-Op and Baseline testing. Cognitive functioning at Baseline (no levetiracetam) was measured one day before administration of levetiracetam and at Pre-Op (with levetiracetam) on the third day after onset of levetiracetam administration. Vertical dotted line represents the clinically meaningful deterioration margin. Bonferroni adjustment was made for six pairwise comparisons. Total Score consists of the domain attention, working memory, memory, and language. Which subtests constitute domains, see methods section

Results for the cognitive domain scores and total scores of cognitive functioning over time are shown in Fig. 3A. Significant p-values were obtained for subtest scores Digit Span (*p* = 0.005), Two Back (*p* = 0.001), Figural Memory (*p* = 0.030), domain scores Working Memory (p < 0.001), performance score Quality (*p* = 0.002) and Total score (*p* = 0.004) (Table 2).

**Table 2:** Estimated marginal means and post-hoc analysis for NeuroCog FX subtest-, domain-, performance- and Total scores for all timepoints from the linear mixed model

	Baseline (*n* = 43)	Pre-Op (*n* = 39)	Post-Op (*n* = 32)	Follow-Up (*n* = 27)			
	*M*_*1*_*, SE*	*M*_*2*_*, SE*	*M3, SE*	*M*_*4*_*, SE*	BIC	*p*	Post-hoc ^**a**^
Digit Span	87.72 (2.25)	92.94 (2.26)	95.10 (1.74)	96.60 (1.68)	1101	.005	1 < 3,4;
TwoBack-Test	91.55 (2.77)	95.43 (3.17)	98.89 (3.17)	108.71 (3.42)	1192	.001	1,2 < 4
Simple Reaction	89.30 (1.95)	88.74 (2.06)	90.31 (1.91)	90.99 (2.14)	1055	.641	-
GoNoGo	90.95 (2.03)	94.29 (1.87)	95.91 (2.38)	97.03 (2.07)	1100	.087	-
Inv GoNoGo	90.72 (1.95)	91.43 (1.92)	93.40 (2.09)	95.20 (2.19)	1099	.297	-
Verbal Memory	86.35 (2.08)	83.76 (1.81)	82.10 (1.86)	85.02 (2.75)	1090	.232	
Figural Memory	90.88 (2.08)	89.05 (1.60)	91.20 (2.08)	94.81 (2.05)	1064	.030	2 < 4
Phonematic Fluency	84.72 (2.37)	83.63 (2.58)	86.31 (2.51)	89.10 (3.00)	1165	.298	-
Psychomotor Speed	90.63 (1.72)	91.57 (1.70)	93.23 (1.89)	94.44 (1.74)	1028	.253	-
Working Memory	89.82 (1.91)	93.73 (2.35)	96.94 (2.09)	102.05 (2.20)	1078	< .001	1 < 3,4; 2 < 4
Memory	88.62 (1.72)	86.39 (1.43)	86.98 (1.74)	90.02 (1.82)	1026	.109	-
Language	84.72 (2.37)	83.63 (2.58)	86.31 (2.51)	89.10 (3.00)	1165	.298	-
Speed	90.63 (1.72)	91.57 (1.70)	93.23 (1.89)	94.44 (1.74)	1028	.253	-
Quality	87.58 (1.55)	88.03 (1.50)	90.00 (1.47)	93.57 (1.64)	995	.002	1,2 < 4
Total	88.28 (1.46)	88.64 (1.43)	91.04 (1.27)	93.85 (1.53)	951	.004	1,2 < 4

Abbreviations: *M* Mean, *SE* standard error, *BIC* Bayes Information Criteria; *p p*-value

Neuropsychological assessment of cognitive functioning was measured at four timepoints (Fig. 1)

^a^Post-hoc analysis (Bonferroni adjusted, six pairwise comparisons) shows significant differences between timepoints. Numbers 1 to 4 refer to respective timepoints: 1 = Baseline, 2 = Pre-Op, 3 = Post-Op, 4 = Follow-Up

### Time interval analysis of cognitive functioning

Mean differences between Baseline (no LEV) and Pre-Op (with LEV) are shown in Fig. 3B. No change in scores was statistically significant, whereas mean differences ranged from -2.59 to 5.22 standard values. The lower bound of the two-sided 95% CI was contained within the critical interval for determining clinically meaningful deterioration in every subtest score. Results for subscores including p-values are given in Supplementary Table 3. Results of mean differences between Baseline and Follow-Up testing are given in Supplementary Table 4. Changes in subtest scores Digit Span, Two Back, Go/No-Go, domain score Working Memory, performance score Quality and Total score are statistically significant.

### HRQoL over time

HRQoL subscale scores and overall score are shown in Supplementary Fig. 5, while LMM model including post-hoc analysis is given in Supplementary Table 5. Significant *p*-values were found for overall score (*p* < 0.001) and all subscale scores, except for social functioning and seizure-worry.

### Correlation between QOLIE31- and NeuroCogFX scores

Pearson correlation between NeuroCogFX Total score and QOLIE31 overall score were not significant at any timepoint (Baseline, r = 0.12, *p* = 0.47; Pre-Op, r = 0.31, *p* = 0.70; Post-Op, r = 0.21; *p* = 0.293; Follow-Up, r = 0.20, *p* = 0.35).

### Subgroup analyses neurrosurgical procedure

Concerning the analysis including time and neurosurgical procedure (stereotacted, neuronavigated biopsy; partial/total resection) as factorial main effect and neurosurgical procedure*time interaction, significant main effects for neurosurgical procedure at α = 0.005 in Total score, in performance score Quality, in domain score Working Memory, in subtest score Two Back, but no significant interaction effect at α = 0.010 in any cognitive score were found (data not shown). Therefore, we performed models again excluding the interaction effect. Results are given in Supplementary Table 6. Significant main effects for neurosurgical procedure in Total Score, in performance score Quality, in domain score Working Memory, and in subtest scores Phonematic Fluency and Digit Span were found; patients who had a stereotacted, neuronavigated biopsy as surgical intervention showed a lower cognitive functioning compared with those who underwent partial or gross-total resection.

## Discussion

The first major finding of this study is that LEV had no negative short-term effect on the cognitive domains studied, as the change observed between Baseline (without LEV) and Pre-op (with LEV) timepoints was not significant in any subtest score. Furthermore, none of the Bonferroni-adjusted 95% confidence intervals of corresponding subtests included the meaningful deterioration margin. These results are consistent with several studies showing that LEV has no negative impact on cognition in both epilepsy patients and healthy subjects [26, 35]. While there are a considerable number of studies investigating the effects of LEV on cognitive function in non-tumor cohorts, we identified only three comparable studies performed with brain tumor patients. However, two of those studies had small sample sizes, used brief and non-specific screening instruments, and included patients who have had seizures before surgery [42, 43]. In their retrospective study in high-grade glioma patients, DeGroot et al. were able to show that LEV was not associated with additional cognitive impairment [34]. Our study is the first trial that confirmed the absence of detrimental effects of LEV on cognitive functioning in a prospective setting including an extensive neurocognitive evaluation.

As second major finding, the postoperative outcome of all subtests showed a trend toward improvement compared to Baseline. Among the multiple studies investigating the change in preoperatively versus postoperative cognition, conflicting results were reported. In a recently published meta-analysis with glioblastoma patients, no statistical analysis could be performed because of the heterogeneity of the cognitive tests, cohorts and time spans analyzed [44]. Our results are in line with a study reporting cognitive improvement postoperatively [45]. In contrast, other studies with mixed histological cohorts reported deteriorated or static cognitive functioning after surgery [46, 47]. In our study, for instance the domain Working Memory, which consists of the subtests Digit Span and Two Back, showed a statistically significant improvement whose magnitude was clinically relevant in our opinion. This effect was presumably resulting from the surgical debulking and the reduction of tumor load, showing thus that working memory is probably very sensitive to changes in tumor volume. Furthermore, in our study, patients who underwent stereotacted, neuronavigated biopsy had statistically significant lower values in some cognitive subtests compared to patients who underwent partial or total resection (Supplementary Results, Supplementary Table 6). Despite the low sample size of the biopsy group, this finding additionally underlines the suspected positive effect of debulking surgery on cognitive functioning. Further exploratory analysis has also shown that left-hemispheric tumor was associated with poorer cognitive functioning, which is consistent with previous findings from the literature (Supplementary Results, Supplementary Table 7) [46, 48].

Means of LEV plasma levels at both timepoints were within the reference level and showed no statistically significant difference, which ensures that LEV was able to exert its effect. The incidence of seizures was 4.65%, which is consistent with reported frequencies in the perioperative setting [11–16]. However, an examination of the efficacy of perioperative anti-seizure medication was beyond the scope of this study.

The course of the HRQoL values showed a similar pattern as with cognitive functioning. There was no significant deterioration between Baseline (no LEV) and Pre-Op (with LEV) testing. Furthermore, most subscores showed a significant change between Baseline and Follow-Up testing. Minimum clinically important change was reported as 11.8 points (95% CI, 9.6–14.0) in epilepsy patients for the overall score, whereas the benchmark for a small change is reported as being 9.8 points (95% CI, 8.0–11.6) [49]. In our sample, change was 10.27 in overall score, which is within the reported confidence interval and exceeds the benchmark for a small clinically meaningful change.

The correlation between cognitive functioning and quality of life reflects a positive relation, although the effect size was in the moderate range (r, 0.12–0.31) and was not significant.

Most reported side effects were graded as mild or moderate, with somnolence being reported most often. The incidence of side effects is consistent with reported frequencies from the literature [19, 21–24].

Hematologic markers were mostly within the normal range (65.8–97.6%), indicating a favorable safety profile.

Several factors contributed to difficulties in screening as well as reasons for exclusion, such as missing inclusion criteria, difficulties in keeping appointments, withdrawn consents and adverse events or deaths after surgery (see Fig. 2). In addition, patients whose cognitive performance was severely impaired had to be excluded from the study, making the feasibility of comprehensive cognitive testing in the perioperative setting of brain tumor surgery rather difficult. However, shorter and probably more feasible cognitive screening instruments, such as the MMSE, may miss important information about cognitive performance.

The following potential implications can be derived from the results of this study; i) perioperative LEV had no detrimental effect on cognitive functioning in brain tumor patients undergoing surgery; ii) no signs of increased hematotoxicity were observed in the perioperative phase; and iii) side effects observed were comparable to the reports in epilepsy patients regarding type, intensity, and frequency. These findings are particularly important for clinicians regarding patient management, as well as for patients at high risk for seizures, where perioperative prophylactic anticonvulsant treatment is deemed necessary.

Additionally, we have shown that particularly working memory improved in the short-term postoperative course. Working memory is an essential component of complex cognitive processes and is involved in all types of information processing and decision-making [26]. According to our study results, clarification and decisions concerning further treatment, as well as important decisions concerning personal life, should therefore probably not be made in the perioperative phase.

This study has limitations: we did not have a control group as our neuro-oncological center routinely applied perioperative AEDs according to internal standard operating procedures; our sample included brain tumors of different histology, as the exact classification was only available after histological examination; neurosurgical procedure included stereotacted, neuronavigated biopsy, partial and gross total resection; in this study, influencing factors—such as possible exercise effects or preoperative anxiety—were not quantitatively controlled.

In conclusion, the present study contributes to the existing literature by showing that perioperative LEV had no detrimental effect on cognitive functioning, quality of life and hematotoxicity in seizure-naive brain tumor patients. Furthermore, we have shown that cognitive functioning, as well as HRQoL, improved meaningful postoperatively.

## Supplementary Information

**Additional file 1:** **Supplementary Material. Supplementary Methods. Supplementary Results. Supplementary Table 1. **Levetiracetam plasma level. **Supplementary Table 2.** Side effects related to study drug levetiracetam. **Supplementary Table 3.** Results of the post-hoc analysis for the mean differences between Pre-Op and Baseline timepoints for NeuroCogFX scores. **Supplementary Table 4.** Results of the post-hoc analysis for the mean differences between Follow-Up and Baseline timepoints for NeuroCogFX scores. **Supplementary Table 5. **Estimated marginal means and post-hoc analysis for QOLIE31 subtest scores and overall score for all timepoints from the linear mixed model. **Supplementary Table 6. **Estimated marginal means for NeuroCog FX subtest-, domain-, performance- and Total scores for all timepoints, subdivided by main effects “time” and “neurosurgical procedure”*. ***Supplementary Table 7. **Estimated marginal means for NeuroCog FXsubtest scores, domain scores, performance scores and Total score for all timepoints, subdivided by maineffects (time, hemisphere) and interaction effect (time*hemisphere). **Supplementary Figure1. **Constitution of different scores of neuropsychological test battery NeuroCog-FX. **Supplementary Figure 2.** Levetiracetam plasma level and occurred seizures. Levetiracetam levels were measured one day before surgery (Pre-Op timepoint, two days after onset) and three days after surgery (Post-Op timepoint, six days after onset).The starting dose of levetiracetam was 2x500 mg on the first day, was escalated to 2 x 1000 mg on the second day and was maintained at this dose for overall nine days. Two patients each, who had a seizure three days post-surgery, are marked with blackpoints.** Supplementary Figure 3. **Severity of hematotoxicity markers in relative percentage. Values were measured one week after surgery. Grading according to National Cancer Institute – Common Terminology Criteria of Adverse Events (CTCAE) v5.0 (Grade 0 = within the normal range, Grade 1 = mild, Grade 2 = moderate, Grade 3 = severe, Grade 4 = life threatening). *n *= 43 in hemoglobin, thrombocytes and leukocytes; *n* = 39 in lymphocytes. Hemoglobin(g/dL): *M*, 12.24; *SD*, 1.43; range, 4.80-50. Thrombocytes(g/L): *M*, 221.5; *SD*, 86.71; range, 51-427. Leukocytes(g/L): *M*, 11.32; *SD*, 3.66; range, 3.5-20.7. Lymphocytes(g/L): *M*, 1.8; *SD*, 1.32; range, 0.3-7.92. **Supplementary Figure 4. **Frequency of patients reporting side effects related to study drug Levetiracetam in absolute percentage across four time points. If a patient reported an adverse reaction more than once and the CTCAE grade differed, the higher severity grade was selected. None of the patients reported side effects regarding abdominal pain, concentration impairment, amnestic aphasia, aggression, anxiety, nightmare, or tinnitus.

## Data Availability

The datasets generated and/or analysed during the current study are not publicly available due privacy restrictions but are available from the corresponding author on reasonable request.
