# Supplementary material for: Perioperative levetiracetam for seizure prophylaxis in seizure-naive brain tumor patients with focus on neurocognitive functioning
Source: BMC Neurol. 2022 Jul 8;22:250. doi: 10.1186/s12883-022-02762-7 (PMC9264633; doi:10.1186/s12883-022-02762-7)
Supplement: Supplementary file 1 — Additional file 1: Supplementary Material. Supplementary Methods. Supplementary Results. Supplementary Table 1. Levetiracetam plasma level. Supplementary Table 2. Side effects related to study drug levetiracetam. Supplementary Table 3. Results of the post-hoc analysis for the mean differences between Pre-Op and Baseline timepoints for NeuroCogFX scores. Supplementary Table 4. Results of the post-hoc analysis for the mean differences between Follow-Up and Baseline timepoints for NeuroCogFX scores. Supplementary Table 5. Estimated marginal means and post-hoc analysis for QOLIE31 subtest scores and overall score for all timepoints from the linear mixed model. Supplementary Table 6. Estimated marginal means for NeuroCog FX subtest-, domain-, performance- and Total scores for all timepoints, subdivided by main effects “time” and “neurosurgical procedure”. Supplementary Table 7. Estimated marginal means for NeuroCog FXsubtest scores, domain scores, performance scores and Total score for all timepoints, subdivided by maineffects (time, hemisphere) and interaction effect (time*hemisphere). Supplementary Figure1. Constitution of different scores of neuropsychological test battery NeuroCog-FX. Supplementary Figure 2. Levetiracetam plasma level and occurred seizures. Levetiracetam levels were measured one day before surgery (Pre-Op timepoint, two days after onset) and three days after surgery (Post-Op timepoint, six days after onset).The starting dose of levetiracetam was 2x500 mg on the first day, was escalated to 2 x 1000 mg on the second day and was maintained at this dose for overall nine days. Two patients each, who had a seizure three days post-surgery, are marked with blackpoints. Supplementary Figure 3. Severity of hematotoxicity markers in relative percentage. Values were measured one week after surgery. Grading according to National Cancer Institute – Common Terminology Criteria of Adverse Events (CTCAE) v5.0 (Grade 0 = within the normal range, Grade 1 = mild, Grade [file 12883_2022_2762_MOESM1_ESM.docx]

**Supplementary material**

**Title:** Perioperative levetiracetam for seizure prophylaxis in seizure-naive brain tumor patients with focus on neurocognitive functioning

**Authors:** Elias Konrath, Franz Marhold, Wolfgang Kindler, Florian Scheichel, Branko Popadic, Katrin Blauensteiner, Bernadette Calabek, Elisabeth Freydl, Michael Weber, Robin Ristl, Katharina Hainz, Camillo Sherif, Stefan Oberndorfer.

**Corresponding author:** Elias Konrath, Department of Neurology, University Hospital St. Pölten, Dunant-Platz 1, 3100, St. Pölten, Austria, +436505162890, [elias.konrath@stpoelten.lknoe.ac.at](mailto:elias.konrath@stpoelten.lknoe.ac.at)

**Supplementary Methods**

**Further analyses**

To evaluate the potential bias caused by patient dropouts, we compared Baseline cognitive mean performances of patients who completed all four assessments with those who dropped out before follow-up testing using independent t-tests. As we had heterogenous histological diagnosis and different neurosurgical procedures in our sample, we looked if the results from the primary analysis of cognitive data changed once we controlled for the influence of the categorical factor variables “histological diagnosis” (low grade=WHO I/II; high grade=WHO III/IV). We have also included interaction terms with time, to account for possible differences in performance over time. Furthermore, we exploratively looked for main and interaction effects for the categorical factor variable “hemisphere” (left/right).

**Supplementary Results**

**Further analysis**

There were no significant differences in cognitive functioning in any subtest-, domain-, performance- or Total Score at Baseline testing between patients who completed all four test assessments and patients who dropped out before Follow-Up testing (p`s>0.05, data not shown).

In further analysis including time and histological diagnosis as factorial main effect and diagnosis*time interaction, no significant main effect at α=0.005 and no significant interaction effect at α=0.010 in any subtest-, domain-, performance- or Total score were found (data not shown). In models where we excluded the interaction effect, no main effect for diagnosis at α=0.005 was found.

In a further explorative analysis including time and hemisphere (left/right) as factorial main effect and hemisphere*time interaction, significant main effects for hemisphere at α=0.005 in subtest scores Two Back, Simple Reaction, Phonematic Fluency, in domain scores Working Memory and Language and in performance score Quality were found (**Supplementary Table 7**). When the tumor was in the left hemisphere, performance was superior in subtest score simple reaction and in performance score speed. In all other scores, performance was superior when the tumor was in the right hemisphere. Interaction effects were found for subtest score Digit Span, where left-hemisphere located tumors showed more improvement over time.

**Supplementary Tables**

| **Supplementary Table 1. Levetiracetam plasma level** | | | | | |
| --- | --- | --- | --- | --- | --- |
|  | ***n*** *(%)* | ***M*** | ***SD*** | ***Mdn*** | ***Range*** |
| Lev-Level Pre-Op (μg/ml) | 37 (100) | 18.16^a^ | 10.58 | 14.44 | 4.80-50.51 |
| Lev-Level Post-Op (μg/ml) | 33 (100) | 18.15^a^ | 12.80 | 15.11 | 5.64-59.65 |

Abbreviations: n = frequency; % = frequency in percentage; M=Mean, SD = standard deviation.;

Mdn = Median.

Levetiracetam levels were measured one day before surgery (Pre-Op timepoint, Dtwo days after onset) and three days after surgery (Post-Op timepoint, Dsix days after onset).

The starting dose of levetiracetam was 2x500 mg on the first day, was escalated to 2 x 1000mg on the second day and was maintained at this dose for overall nine days.

^a^Levetiracteam levels Pre-Op and Post-Op do not differ statistically significantly (p>0.05).

|  | **Supplementary Table 2.** **Side effects related to study drug levetiracetam** | | | | | | | |
| --- | --- | --- | --- | --- | --- | --- | --- | --- |
|  | **Across evaluation period** | | **Timepoint** | | | | | |
| **Category** | **Patients, *n* (%)**  **(*n* = 42)^a^** | **Frequency of side effects^b^ *n* (%)** | **Pre-Op,**  ***n* (%)**  **(*n* = 42)** | | **Post-Op,**  ***n* (%)**  **(*n* = 32)** | | **Follow-Up,**  ***n* (%)**  **(*n* = 27)** | |
|  |  |  | Number of patients/side effects^c,d^ | CTCAE  Grading^e^ | Number of patients/side effects^c,d^ | CTCAE  Grading^e^ | Number of patients/side effects^c,d^ | CTCAE  Grading^e^ |
| Somnolence | 12 (28.6%) | 20 (47.6%) | 10 (23.8%) | 5 (50%) Mild  2 (20%) Moderate  3 (30%) Severe  0 (0%) Serious | 7 (21.9%) | 3 (43%) Mild  2 (28%) Moderate  2 (28%) Severe  0 (0%) Serious | 3 (11.1%) | 1 (33%) Mild  1 (33%) Moderate  0 (0%) Severe  1 (33%) Serious |
| Vertigo | 3 (7.1%) | 4 (9.5%) | 3 (7.1%) | 1 (33%) Mild  1 (33%) Moderate  1 (33%) Severe | 1 (3.1%) | 1 (100%) Mild  0 (0%) Moderate  0 (0%) Severe | 0 (0%) | ––– |
| Subj. Mem. Imp. | 3 (7.1%) | 5 (11.9%) | 2 (4.8%) | 2 (100%) Mild  0 (0%) Moderate | 2 (6.3%) | 1 (50%) Mild  1 (50%) Moderate | 1 (3.7%) | 0 (0%) Mild  1 (100%) Moderate |
| Cephalea | 2 (4.8%) | 3 (7.1%) | 2 (4.8%) | 2 (100%) Mild | 1 (3.1%) | 1 (100%) Mild | 0 (0%) | ––– |
| Depression | 2 (4.8%) | 3 (7.1%) | 1 (2.4%) | 1 (100%) Moderate | 1 (3.1%) | 1 (100%) Moderate | 1 (3.7%) | 1 (100%) Moderate |
| Exanthem | 2 (4.8%) | 3 (7.1%) | 0 (0%) | ––– | 2 (6.3%) | 2 (100%) Moderate | 1 (3.7%) | 1 (100%) Moderate |
| Nausea | 1 (2.4%) | 1 (2.4%) | 1 (2.4%) | 1 (100%) Moderate | 0 (0%) | ––– | 0 (0%) | ––– |
| Nasopharyngitis | 1 (2.4%) | 1 (2.4%) | 1 (2.4%) | 1 (100%) Mild | 0 (0%) | ––– | 0 (0%) | ––– |
| Diarrhea | 1 (2.4%) | 1 (2.4%) | 1 (2.4%) | 1 (100%) Moderate | 0 (0%) | ––– | 0 (0%) | ––– |
| Opthalmalgia | 1 (2.4%) | 1 (2.4%) | 0 (0%) | ––– | 0 (0%) | ––– | 1 (3.7%) | 1 (100%) Mild |
| Total | 21 (48.8%) | 42 (100%) | Side effects: 21 (48.8%)^c^  Patients:  16 (37.2%)^d^ |  | Side effects: 14 (33.3%)^c^  Patients:  12 (27.9%)^d^ |  | Side effects:  7 (16.7%)^c^  Patients:  5 (18.5%)^d^ |  |

Abbreviations: n = frequency.

Grading according to National Cancer Institute – Common Terminology Criteria of Adverse Events (CTCAE) v5.0 (Grade 0 = within the normal range, Grade 1 = mild, Grade 2 = moderate, Grade 3 = severe, Grade 4 = life threatening).

Self-perceived side effects were measured at four timepoints: one day before administration of levetiracetam (Baseline/no levetiracetam), on the third day after onset of levetiracetam administration (Pre-Op/with levetiracetam), four to six days after surgery (Post-Op/with levetiracetam) and in the context of a follow-up examination three weeks after surgery (Follow-Up/no levetiracetam).

^a^Numbers in this column refer to frequency of all patients regardless of timepoint (n = 42).

^b^Numbers in this column refer to frequency of all side effects reported across four timepoints (n = 42).

^c^Number refers to frequency of all side effects across four timepoints (n = 42).

^d^Number refers to frequency of affected patients at respective timepoint (Pre-Op: n = 42, Post-Op: n = 32, Follow-Up: n = 27).

^e^Numbers in this column refers to frequency of respective the reported side effect at a given timepoint.

**Supplementary Table 3.** **Results of the post-hoc analysis for the mean differences between Pre-Op and Baseline timepoints for NeuroCogFX scores**

|  | ***M_2_-M_1_*** | ***SE*** | **95% *CI* adj.** | ***p* adj.** | **95% *CI*** | ***p*** |
| --- | --- | --- | --- | --- | --- | --- |
| Digit Span | 5.22 | 1.99 | -0.30, 10.75 | .074 | 1.20, 9.24 | 0.012 |
| Two Back | 3.88 | 3.11 | -4.77, 12.52 | 1.0 | -2.41, 10,16 | 0.220 |
| Simple Reaction | -0.56 | 1.64 | -5.1, 4.0 | 1.0 | -3.86, 2.75 | 0.736 |
| Go/No-Go | 3.34 | 1.81 | -1.70, 8.37 | .438 | -0.32, 7.00 | 0.073 |
| Inv. Go/No-Go | 0.71 | 1.90 | -5.98, 4.56 | 1.0 | -3.13, 4.55 | 0.711 |
| Verbal Memory | -2.59 | 2.02 | -8.18, 2.99 | 1.0 | -6.66, 1.48 | 0.205 |
| Figural Memory | -1.83 | 1.86 | -7.02, 3.36 | 1.0 | -5.61, 1.95 | 0.333 |
| Phonematic Fluency | -1.09 | 2.55 | -8.19, 6.01 | 1.0 | -6.25, 4.08 | 0.672 |
| Attention | 0.94 | 1.32 | -2.73, 4.62 | 1.0 | -1.73, 3.62 | 0.481 |
| Working Memory | 3.91 | 2.01 | -1.69, 9.51 | .353 | -0.15, 7.98 | 0.059 |
| Memory | -2.23 | 1.58 | -6.63, 2.17 | 1.0 | -5.43, 0.98 | 0.167 |
| Language | -1.09 | 2.55 | -8.19, 6.01 | 1.0 | -6.25, 4.08 | 0.672 |
| Speed | 0.94 | 1.32 | -2.73, 4.62 | 1.0 | -1.73, 3.62 | 0.481 |
| Quality | 0.45 | 1.32 | -3.22, 4.12 | 1.0 | -2.22, 3.12 | 0.735 |
| Total | 0.36 | 1.25 | -3.84, 3.13 | 1.0 | -2.17, 2.88 | 0.777 |

Abbreviations: M = mean; SE = standard error; CI = confidence interval; p = p-value; adj. = Bonferroni adjusted.

Estimated marginal means and corresponding 95% confidence intervals in standard value points (M = 100, SD = 10) for cognitive subtest scores, domain scores, performance scores and Total score from difference between Pre-Op (M_2_) and Baseline (M_1_) timepoints. 95% CI and p-values are given Bonferroni adjusted (six pairwise comparisons) and unadjusted.

**Supplementary Table 4.** **Results of the post-hoc analysis for the mean differences between Follow-Up and Baseline timepoints for NeuroCogFX scores**

|  | ***M_4_-M_1_*** | ***SE*** | **95%*CI* adj.** | ***p* adj.** | **95% *CI*** | ***p*** |
| --- | --- | --- | --- | --- | --- | --- |
| Digit Span | 8.88 | 2.39 | 2.23, 15.52 | .004 | 4.04, 13.71 | .001 |
| Two Back | 17.16 | 3.77 | 6.54, 27.78 | <.001 | 9.47, 24.85 | <.001 |
| Simple Reaction | 1.70 | 2.38 | -4.93, 8.33 | 1.0 | -3.13, 6.52 | .480 |
| GoNoGo | 6.08 | 2.53 | -0.97, 13.13 | .128 | 0.96, 11.20 | .021 |
| Inv GoNoGo | 4.48 | 2.53 | -2.59, 11.56 | .509 | -0.65, 9.62 | .085 |
| Verbal Memory | -1.33 | 2.35 | -7.99, 5.33 | 1.0 | -6.14, 3.48 | .577 |
| Figural Memory | 3.93 | 2.62 | -3.37, 11.22 | .850 | -1.37, 9.23 | .142 |
| Phonematic Fluency | 4.38 | 2.91 | -3.85, 12.60 | .859 | 1.57, 10.32 | .143 |
| Attention | 3.81 | 1.94 | -1.59, 9.22 | .341 | -0.12, 7.75 | .057 |
| Working Memory | 12.23 | 2.19 | 6.09, 18.37 | <.001 | 7.78, 16.68 | <.001 |
| Memory | 1.40 | 1.83 | -3.73, 6.54 | 1.0 | -2.32, 5.13 | .448 |
| Language | 4.38 | 2.91 | -3.85, 12.60 | .859 | 1.57, 10.32 | .143 |
| Speed | 3.81 | 1.94 | -1.59, 9.22 | .341 | -0.12, 7.75 | .057 |
| Quality | 5.99 | 1.55 | 1.64, 10.34 | .003 | 2.84, 9.14 | .001 |
| Total | 5.56 | 1.59 | 1.09, 10.04 | .008 | 2.33, 8.80 | .001 |

Abbreviations: M = mean; SE = standard error; CI = confidence interval; p = p-value; adj. = Bonferroni adjusted.

Estimated marginal means and corresponding 95% confidence intervals in standard value points (M = 100, SD = 10) for cognitive subtest scores, domain scores, performance scores and Total score from difference between Follow Up (M_4_) and Baseline (M_1_) timepoints. 95% CI and p-values are given Bonferroni adjusted (six pairwise comparisons) and unadjusted.

**Supplementary Table 5.** **Estimated marginal means and post-hoc analysis for QOLIE31 subtest scores and overall score for all timepoints from the linear mixed model**

|  | **Baseline**  **(*n* = 41)** | **Pre-Op**  **(*n* = 38)** | **Post-Op**  **(*n* = 29)** | **Follow-Up (*n* = 24)** |  |  |  |
| --- | --- | --- | --- | --- | --- | --- | --- |
|  | ***M_1_, SE*** | ***M_2_, SE*** | ***M_3_, SE*** | ***M_4_, SE*** | **BIC** | ***p*** | **Post-hoc^a^** |
| Seizure Worry | 86.64 (3.13) | 90.61 (3.47) | 87.03 (3.67) | 92.73 (2.85) | 1097 | .093 | - |
| Overall QoL | 66.77 (2.71) | 62.11 (3.22) | 72.10 (3.29) | 78.80 (2.92) | 1132 | <.001 | 2 < 3, 4; 1 < 4 |
| Emotional Well-Being | 72.26 (3.37) | 76.06 (3.42) | 80.93 (2.80) | 87.49 (2.29) | 1129 | .001 | 1,2 < 4 |
| Energy/Fatigue | 61.12 (3.64) | 62.13 (4.17) | 75.15 (3.33) | 78.43 (2.76) | 1188 | .001 | 1,2 < 3,4 |
| Cognitive Functioning | 77.53 (3.18) | 79.28 (3.81) | 81.39 (3.44) | 89.42 (3.20) | 1167 | .018 | 1 < 4 |
| Medication Effects | 85.25 (4.07) | 90.09 (3.06) | 90.60 (4.31) | 98.93 (1.35) | 1146 | .003 | 1 < 4 |
| Social Functioning | 79.14 (2.88) | 76.31 (3.86) | 76.49 (5.01) | 79.12 (4.19) | 1199 | .772 | - |
| Overall Score | 74.62 (2.13) | 74.60 (2.80) | 79.46 (2.54) | 84.89 (1.99) | 1045 | <.001 | 1,2,3 < 4 |

Abbreviations: M = mean; SE = standard error; BIC = Bayes Information Criteria; p = p-value.

Scores range from 0 to 100, with higher scores reflecting better quality of life.

Health-related quality of life was measured at four timepoints (**Figure 2**).

^a^Post-hoc analysis (Bonferroni adjusted, six pairwise comparisons) shows significant differences between timepoints. Numbers 1 to 4 refer to respective timepoints: 1 = Baseline, 2 = Pre-Op, 3 = Post-Op, 4 = Follow-Up.

**Supplementary Table 6. Estimated marginal means for NeuroCog FX subtest-, domain-, performance- and Total scores for all timepoints, subdivided by main effects “time” and “neurosurgical procedure”**

|  | **Baseline**  **(*n* = 42)** | **Pre-Op**  **(*n* = 38)** | **Post-Op**  **(*n* = 32)** | **Follow-Up**  **(*n* = 27)** | **Neurosurgical procedure**  **Biopsy Partial/Total**  **(*n* = 7) (*n* = 35)** | | ***p*** | |
| --- | --- | --- | --- | --- | --- | --- | --- | --- |
|  | ***M (SE)*** | ***M (SE)*** | ***M (SE)*** | ***M (SE)*** | ***M (SE)*** | ***M (SE)*** | **Time** | **Procedere** |
| Digit Span | 84.26 (2.59) | 88.82 (2.58) | 91.26 (2.36) | 92.49 (2.42) | 84.56 (3.97) | 93.85 (1.53) | .011 | .034 |
| Two Back Test | 87.68 (3.63) | 91.51 (4.03) | 94.92 (3.96) | 104.29 (4.34) | 89.07 (6.30) | 100.13 (2.54) | .001 | .109 |
| Simple Reaction | 89.41 (2.56) | 88.85 (2.68) | 90.47 (2.60) | 91.16 (2.82) | 90.24 (4.42) | 89.70 (1.85) | .631 | .909 |
| Go/NoGo | 92.25 (2.56) | 96.03 (2.48) | 97.55 (2.92) | 98.99 (2.81) | 98.58 (4.27) | 93.83 (1.73) | .055 | .306 |
| Invers Go/NoGo | 90.03 (2.45) | 90.91 (2.50) | 92.88 (2.63) | 94.78 (2.85) | 91.81 (4.10) | 92.49 (1.62) | .262 | .877 |
| Verbal Memory | 84.25 (2.42) | 81.13 (2.15) | 80.01 (2.34) | 82.93 (2.98) | 79.31 (3.47) | 84.85 (1.67) | .196 | .136 |
| Figural Memory | 88.70 (2.53) | 86.47 (2.20) | 88.70 (2.56) | 92.08 (2.66) | 85.86 (3.95) | 92.11 (1.63) | .030 | .147 |
| Phonematic Fluency | 81.28 (2.85) | 80.00 (3.13) | 82.15 (3.05) | 85.06 (3.55) | 76.07 (4.78) | 88.17 (2.02) | .406 | .023 |
| Psychomotor Speed | 90.95 (2.24) | 91.98 (2.26) | 93.68 (2.42) | 95.03 (2.40) | 93.79 (3.88) | 92.02 (1.61) | .215 | .675 |
| Working Memory | 85.83 (2.41) | 89.41 (2.81) | 92.75 (2.61) | 97.60 (2.77) | 85.79 (4.26) | 97.01 (1.78) | <.001 | .019 |
| Memory | 86.45 (2.08) | 83.76 (1.85) | 84.54 (2.15) | 87.55 (2.22) | 82.59 (3.28) | 88.57 (1.41) | .079 | .096 |
| Language | 81.28 (2.85) | 80.00 (3.13) | 82.15 (3.05) | 85.06 (3.55) | 76.07 (4.78) | 88.17 (2.02) | .406 | .023 |
| Speed | 90.95 (2.24) | 91.98 (2.26) | 93.68 (2.42) | 95.03 (2.40) | 93.79 (3.88) | 92.02 (1.61) | .215 | .675 |
| Quality | 84.43 (1.87) | 84.57 (1.89) | 86.43 (1.88) | 90.05 (2.03) | 81.59 (3.10) | 91.15 (1.30) | .003 | .006 |
| Total | 85.58 (1.77) | 85.67 (1.79) | 88.02 (1.68) | 90.78 (1.91) | 83.46 (2.91) | 91.56 (1.23) | .006 | .013 |

Abbreviations: n = frequency; M = mean; SE = standard error; p = p-value; Procedere = neurosurgical procedere; Biopsy = stereotacted, neuronavigated biopsy.

Neuropsychological assessment of cognitive functioning was measured at four timepoints (**Figure 1**).

**Supplementary Table 7. Estimated marginal means for NeuroCog FX subtest scores, domain scores, performance scores and Total score for all timepoints, subdivided by main effects (time, hemisphere) and interaction effect (time*hemisphere)**

|  |  | **Baseline**  **(*n* = 43)** | **Pre-Op**  **(*n* = 39)** | **Post-Op**  **(*n* = 39)** | **Follow-Up**  **(*n* = 27)** | **Hemisphere**  **Left Right**  **(*n* = 19) (*n* = 24)** | | ***p*** | | |
| --- | --- | --- | --- | --- | --- | --- | --- | --- | --- | --- |
|  |  | ***M (SE)*** | ***M (SE)*** | ***M (SE)*** | ***M (SE)*** | ***M (SE)*** | ***M (SE)*** | **Time** | **Hem.** | **Int.** |
| Digit Span | left | 80.11 (3.03) | 90.28 (3.48) | 93.55 (2.93) | 96.62 (2.77) | 90.14 (2.27) | 95.48 (1.92) | .001 | .081 | .024 |
|  | right | 93.75 (2.69) | 95.11 (2.97) | 96.33 (2.20) | 96.72 (2.16) |  |  |  |  |  |
| Two Back Test | left | 87.81 (4.20) | 90.82 (4.84) | 95.22 (5.18) | 98.30 (5.11) | 93.04 (3.56) | 102.64 (3.01) | .001 | .046 | .440 |
|  | right | 94.46 (3.68) | 98.97 (4.16) | 101.56 (4.09) | 115.58 (4.0) |  |  |  |  |  |
| Simple Reaction | left | 94.58 (2.72) | 93.13 (3.07) | 93.55 (3.10) | 95.05 (3.43) | 94.08 (2.48) | 86.59 (2.08) | .615 | .026 | .745 |
|  | right | 85.02 (2.45) | 85.33 (2.63) | 87.84 (2.39) | 88.17 (2.68) |  |  |  |  |  |
| Go/NoGo | left | 93.63 (3.04) | 94.38 (2.93) | 102.23 (3.79) | 97.33 (3.45) | 96.90 (2.49) | 92.99 (2.10) | .066 | .238 | .121 |
|  | right | 88.83 (2.70) | 94.08 (2.49) | 92.12 (2.96) | 96.93 (2.68) |  |  |  |  |  |
| Invers Go/NoGo | left | 94.74 (2.84) | 92.66 (3.0) | 95.22 (3.43) | 96.09 (3.65) | 94.68 (2.31) | 91.05 (1.95) | .385 | .237 | .543 |
|  | right | 87.54 (2.53) | 90.35 (2.54) | 91.87 (2.67) | 94.45 (2.83) |  |  |  |  |  |
| Verbal Memory | left | 84.00 (3.13) | 83.38 (2.84) | 81.93 (3.11) | 82.85 (4.46) | 83.04 (2.50) | 85.32 (2.10) | .310 | .490 | .715 |
|  | right | 88.21 (2.79) | 84.11 (2.40) | 82.33 (2.41) | 86.62 (3.56) |  |  |  |  |  |
| Figural Memory | left | 89.42 (3.16) | 87.57 (2.49) | 88.77 (3.31) | 94.37 (3.39) | 90.03 (2.35) | 92.56 (1.99) | .038 | .417 | .905 |
|  | right | 92.04 (2.81) | 90.14 (2.11) | 92.86 (2.67) | 95.18 (2.65) |  |  |  |  |  |
| Phonematic Fluency | left | 80.00 (3.47) | 75.34 (3.61) | 79.80 (3.94) | 84.32 (4.82) | 79.84 (2.79) | 90.41 (2.33) | .290 | .006 | .662 |
|  | right | 88.46 (3.09) | 89.74 (3.04) | 90.76 (2.99) | 92.67 (3.79) |  |  |  |  |  |
| Psychomotor Speed | left | 94.32 (2.50) | 93.59 (2.60) | 97.12 (3.01) | 96.05 (2.85) | 95.27 (2.22) | 90.36 (1.89) | .275 | .099 | .532 |
|  | right | 87.71 (2.23) | 89.91 (2.23) | 90.52 (2.39) | 93.29 (2.24) |  |  |  |  |  |
| Working Memory | left | 84.30 (2.71) | 90.06 (3.56) | 94.37 (3.37) | 96.99 (3.43) | 91.43 (2.55) | 98.92 (2.17) | <.001 | .031 | .469 |
|  | right | 94.10 (2.37) | 96.64 (3.08) | 99.19 (2.66) | 105.76 (2.70) |  |  |  |  |  |
| Memory | left | 86.71 (2.59) | 85.45 (2.24) | 85.80 (2.84) | 88.55 (2.96) | 86.63 (2.07) | 89.04 (1.75) | .150 | .380 | .957 |
|  | right | 90.13 (2.31) | 87.12 (1.89) | 87.84 (2.24) | 91.08 (2.35) |  |  |  |  |  |
| Language | left | 80.00 (3.47) | 75.34 (3.61) | 79.80 (3.94) | 84.32 (4.82) | 79.84 (2.79) | 90.41 (2.33) | .290 | .006 | .662 |
|  | right | 88.46 (3.09) | 89.74 (3.04) | 90.76 (2.99) | 92.67 (3.79) |  |  |  |  |  |
| Speed | left | 94.32 (2.50) | 93.59 (2.60) | 97.12 (3.01) | 96.05 (2.85) | 95.27 (2.22) | 90.36 (1.89) | .275 | .099 | .532 |
|  | right | 87.71 (2.23) | 89.91 (2.23) | 90.52 (2.39) | 93.29 (2.24) |  |  |  |  |  |
| Quality | left | 83.37 (2.19) | 83.84 (2.15) | 86.32 (2.28) | 89.75 (2.54) | 85.82 (1.75) | 92.84 (1.48) | .004 | .004 | .980 |
|  | right | 90.92 (1.94) | 91.30 (1.83) | 92.70 (1.79) | 96.45 (2.02) |  |  |  |  |  |
| Total | left | 85.85 (2.19) | 86.12 (2.15) | 88.60 (1.98) | 91.34 (2.45) | 88.0 (1.73) | 92.29 (1.48) | .008 | .066 | .999 |
|  | right | 90.17 (1.92) | 90.59 (1.85) | 92.78 (1.60) | 95.62 (1.93) |  |  |  |  |  |

Abbreviations: n = frequency; M = mean; SE = standard error; p=p-value; Hem. = hemisphere; Int. = interaction.

Neuropsychological assessment of cognitive functioning was measured at four timepoints (**Figure 1**).

**Supplementary Figures**

**Supplementary Figure 1. Constitution of different scores of neuropsychological test battery NeuroCog-FX**


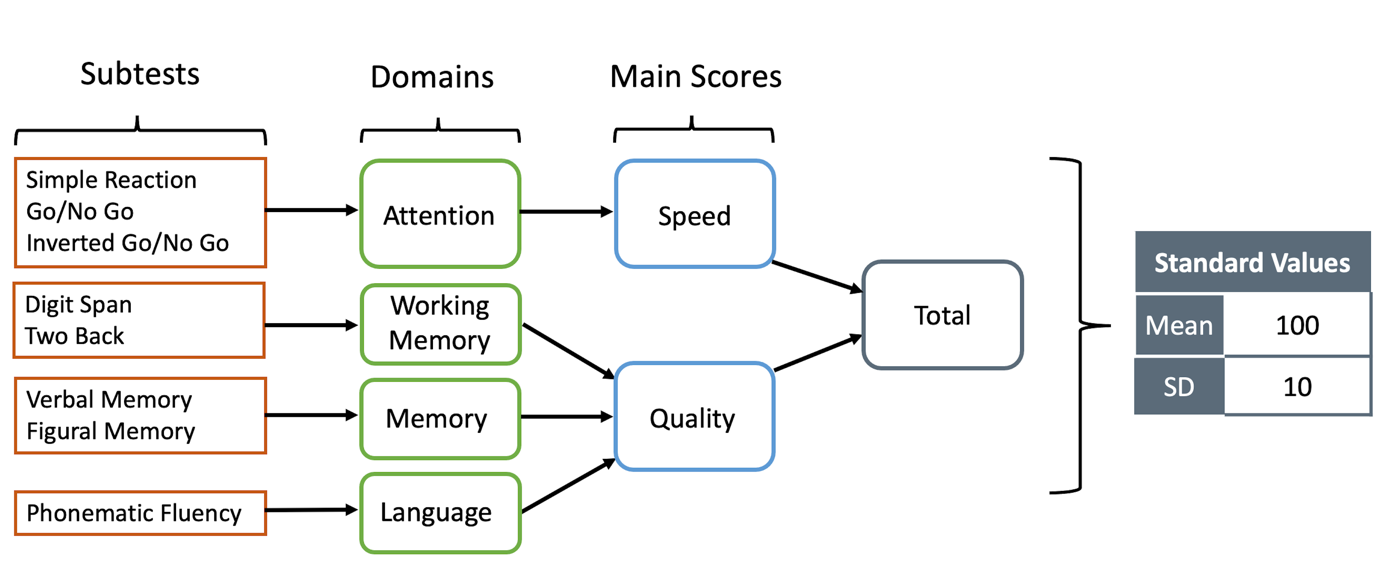


**Supplementary Figure 2. Levetiracetam plasma level and occurred seizures**

Levetiracetam levels were measured one day before surgery (Pre-Op timepoint, two days after onset) and three days after surgery (Post-Op timepoint, Dsix days after onset). The starting dose of levetiracetam was 2x500 mg on the first day, was escalated to 2 x 1000 mg on the second day and was maintained at this dose for overall nine days.

Two patients each, who had a seizure three days post-surgery, are marked with black points.


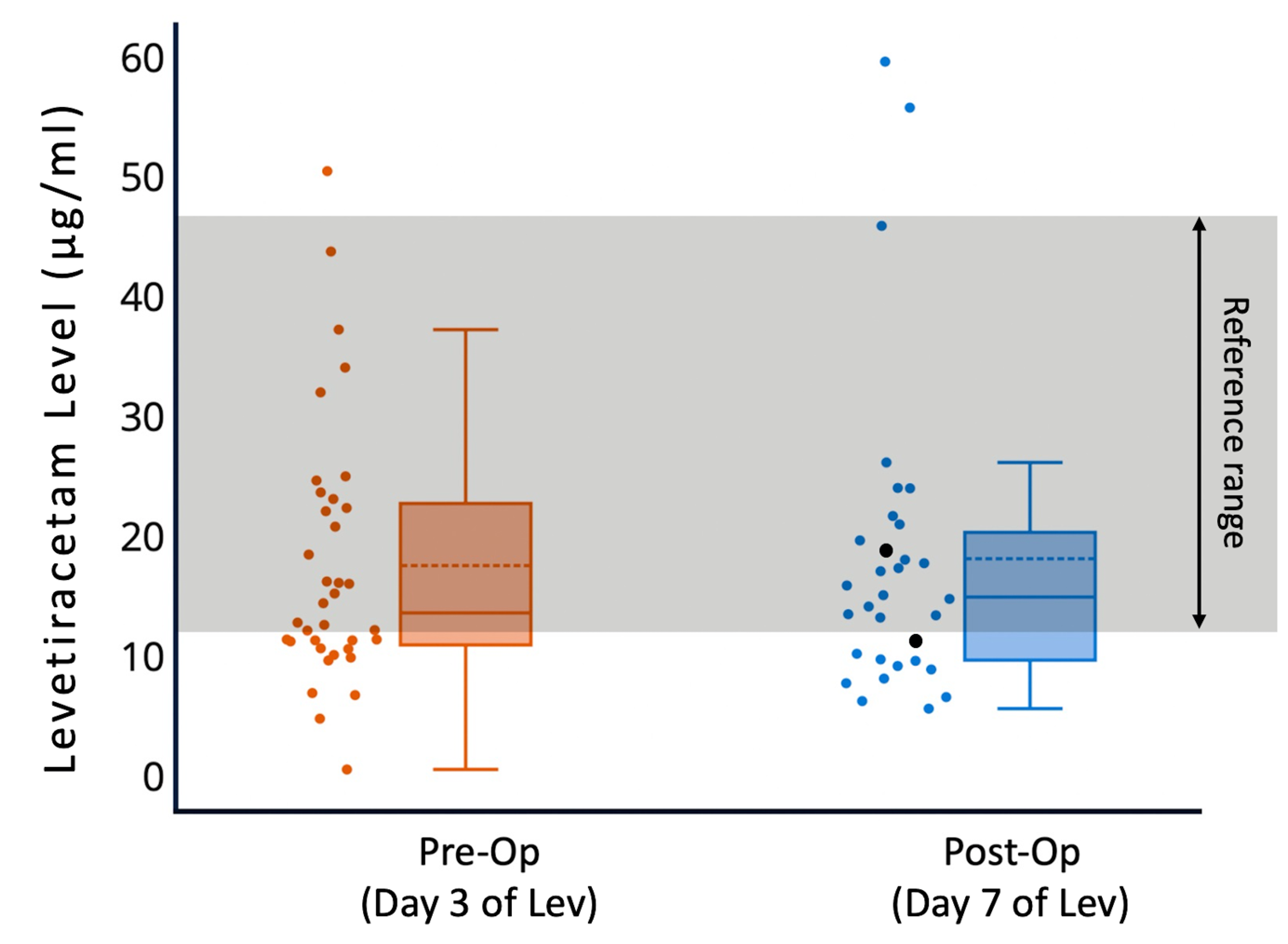


**Supplementary Figure 3. Severity of hematotoxicity markers in relative percentage**

Values were measured one week after surgery. Grading according to National Cancer Institute – Common Terminology Criteria of Adverse Events (CTCAE) v5.0 (Grade 0 = within the normal range, Grade 1 = mild, Grade 2 = moderate, Grade 3 = severe, Grade 4 = life threatening).

*n* = 43 in hemoglobin, thrombocytes and leukocytes; *n* = 39 in lymphocytes.

Hemoglobin (g/dL): *M*, 12.24; *SD*, 1.43; range, 4.80-50.

Thrombocytes (g/L): *M*, 221.5; *SD*, 86.71; range, 51-427.

Leukocytes (g/L): *M*, 11.32; *SD*, 3.66; range, 3.5-20.7.

Lymphocytes (g/L): *M*, 1.8; *SD*, 1.32; range, 0.3-7.92.


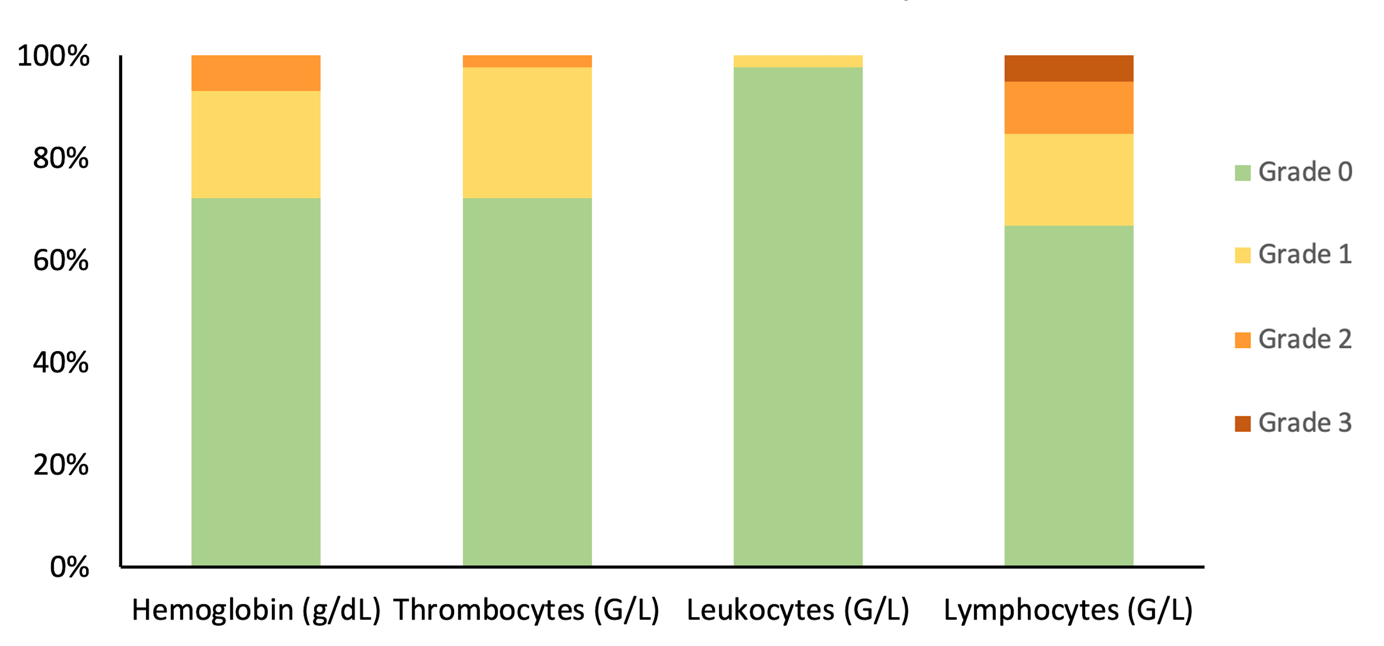


**Supplementary Figure 4. Frequency of patients reporting side effects related to study drug levetiracetam in absolute percentage across four time points**

If a patient reported an adverse reaction more than once and the CTCAE grade differed, the higher severity grade was selected. None of the patients reported side effects regarding abdominal pain, concentration impairment, amnestic aphasia, aggression, anxiety, nightmare, or tinnitus.


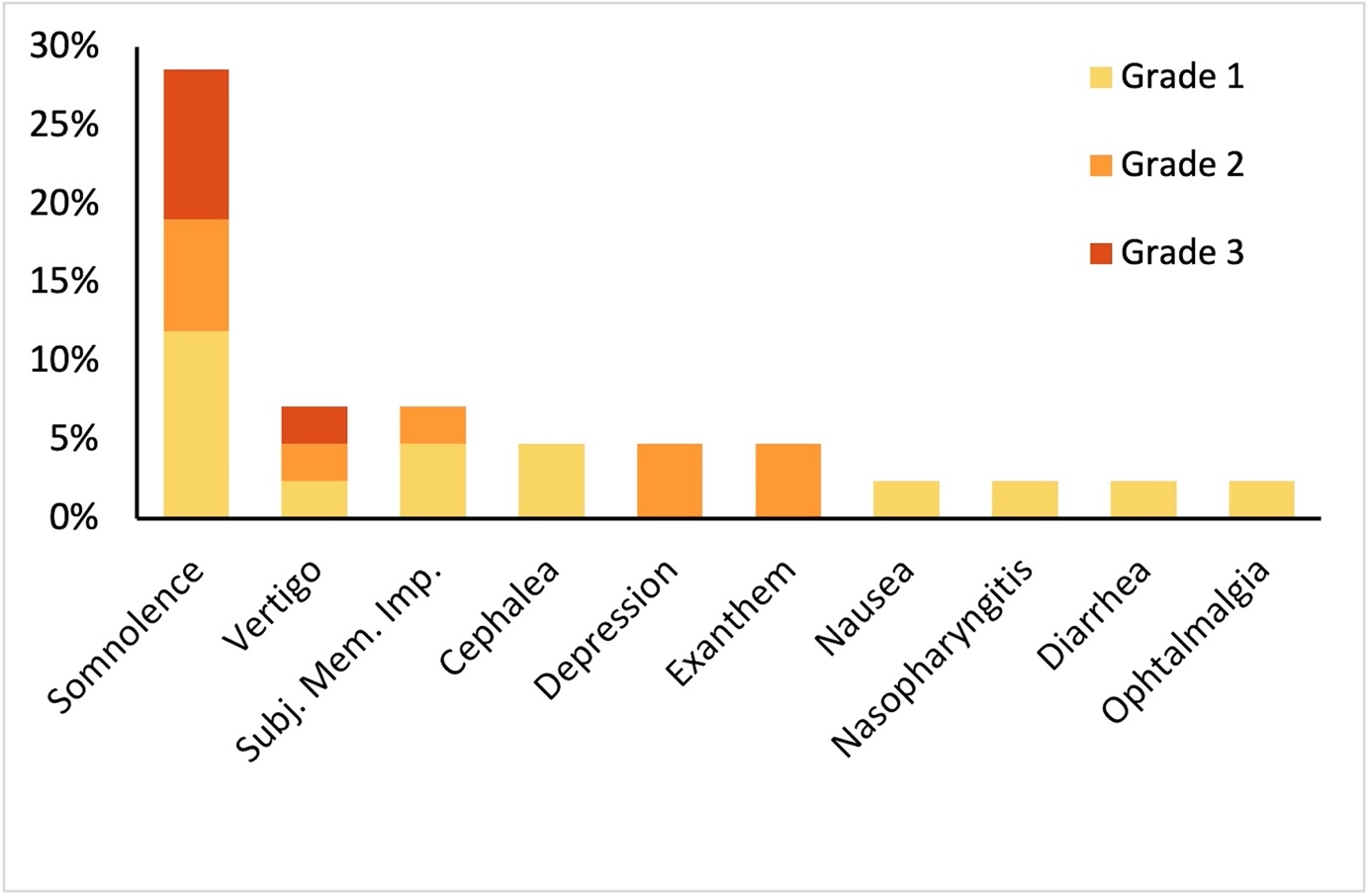


**Supplementary Figure 5.** **Estimated marginal means and corresponding standard error bars in standard value points across the four timepoints for the health-related quality of life subscale scores and overall score**

Health-related quality of life was measured at four time points (**Figure 1**).


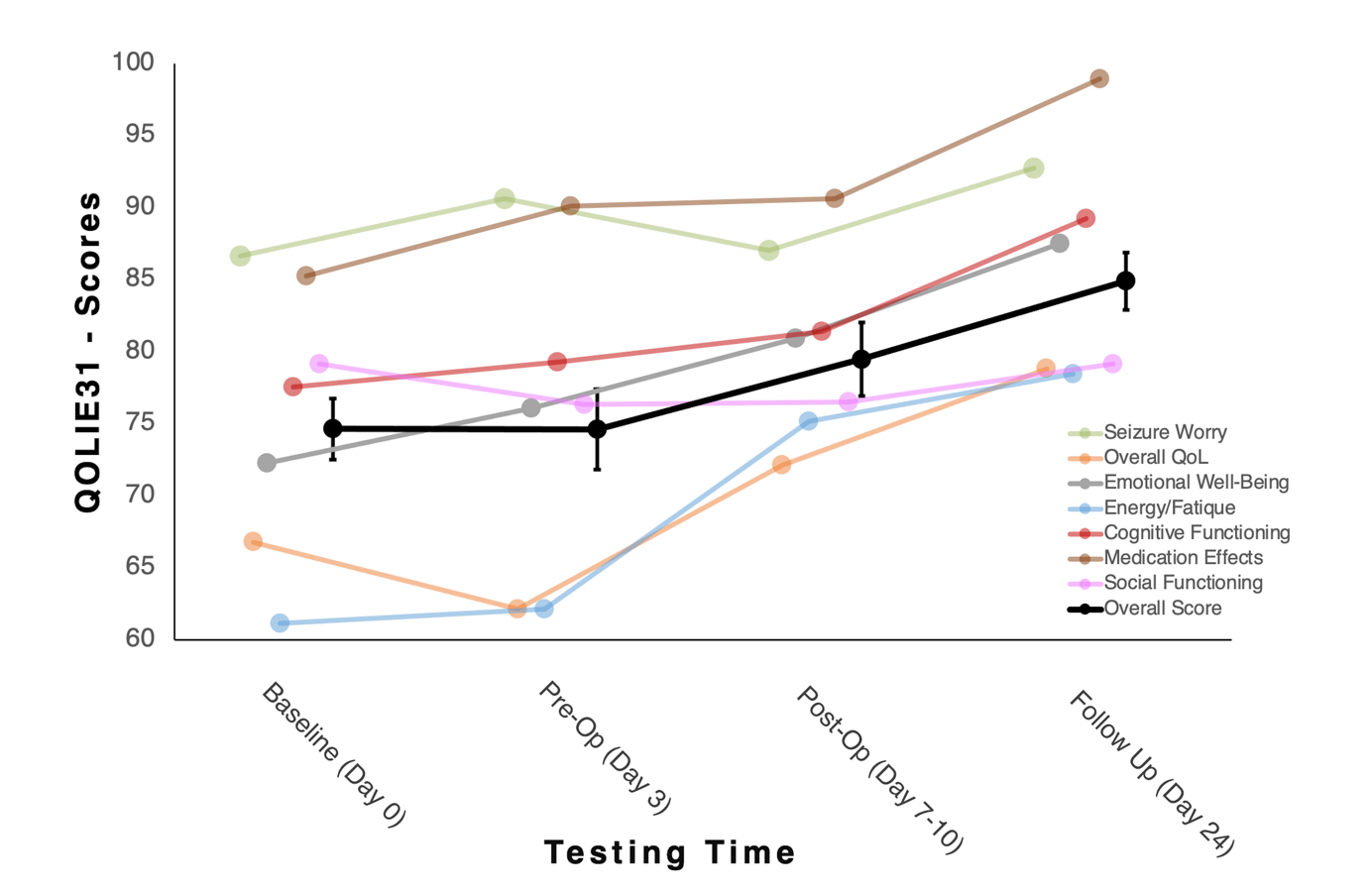


**Supplementary References**
